# Exploring the Association of Helicobacter pylori With Anti-intrinsic Factor and Anti-parietal Cell Antibodies in Pernicious Anemia: A Systematic Review

**DOI:** 10.7759/cureus.45887

**Published:** 2023-09-25

**Authors:** Akhil Allakky

**Affiliations:** 1 Internal Medicine, The Health Herald, Orange, USA

**Keywords:** vitamin b12 deficiency, immunopathology, autoantibodies, atrophic gastritis, clinical hematology, helicobacter pylori and pernicious anemia

## Abstract

Pernicious anemia, historically tied to vitamin B12 malabsorption due to intrinsic factor secretion impairment, has evolved in understanding, especially concerning its association with autoimmune gastritis. This systematic review dives deep into the multifaceted relationship between *Helicobacter pylori (H. pylori)* infection, autoimmune gastritis, and the presence of anti-intrinsic factors and anti-parietal cell antibodies. Comprehensive database searches revealed a higher prevalence of *H. pylori *infection in pernicious anemia patients, with some studies suggesting a consequential increase in the aforementioned antibodies. Interestingly, eradication of *H. pylori* displayed potential therapeutic effects; patients showcased reductions in antibody titers, improved histopathological findings, and reversion of atrophic changes in gastric corpus. Such outcomes highlight the conceivable benefits of considering *H. pylori* infection during the evaluation and management of pernicious anemia and autoimmune gastritis. However, disparities across studies make direct comparisons challenging. It's essential to approach the potential role of *H. pylori *in these conditions with caution. Further research is warranted to cement conclusions and refine clinical management strategies. This review seeks to prompt new investigative avenues into the intricate link between *H. pylori,* autoimmune gastritis, and pernicious anemia, ultimately enhancing patient care.

## Introduction and background

Pernicious anemia also known as Biermer anemia or Addison-Biermer disease was first described in 1822 in Edinburgh [1,2]. Pernicious anemia is a condition characterized by the body's inability to effectively absorb vitamin B12 from the stomach, which is primarily caused by the presence of antibodies against intrinsic factor (IF) and parietal cells [3]. These specific antibodies, referred to as anti-IF and anti-parietal antibodies, serve as highly indicative markers for diagnosing pernicious anemia [3].

The development of these antibodies can be attributed to a related condition known as autoimmune gastritis (AIG), characterized by the presence of T-helper1 (Th1) CD4 T-cells that target the gastric H/k ATPase found in the gastric mucosa [4]. Consequently, this autoimmune response leads to the destruction of the parietal cells within the gastric glands, which play a crucial role in producing gastric acid and IF [4].

*Helicobacter pylori* *(H. pylori)*, a bacterium associated with chronic gastritis and peptic ulcers, has also been identified as a potential factor in the development of pernicious anemia [5]. While the primary etiology of pernicious anemia is related to anti-IF and anti-parietal antibodies, studies have investigated the possible involvement of *H. pylori *in the pathogenesis of this condition. Although there is no definite theory explaining the pathogenesis, a commonly discussed theory is that the destruction of gastric parietal and antral cells which leads to AIG, also causes an impediment in vitamin B12 transport [5,6].

A higher prevalence of *H. pylori* infection in patients with pernicious anemia compared to control groups has been widely reported, suggesting a possible link between the two [6]. Additionally, *H. pylori *eradication has been associated with improvements in gastric histology and a decrease in anti-parietal cell antibodies, further supporting the hypothesis of an association [7].

Despite these findings, the exact mechanisms underlying the potential association between *H. pylori *and pernicious anemia are not fully understood. It has been proposed that *H. pylori* infection may induce chronic inflammation and immune responses, which could contribute to the production of anti-parietal antibodies [8].

To date, the relationship between anti-IF and anti-parietal antibodies and *H. pylori* infection in the context of pernicious anemia remains an area of ongoing research. The precise mechanisms and causality require further investigation to establish a definitive link between these factors. Understanding the potential association between *H. pylori* infection and pernicious anemia is of clinical importance, as it may provide insights into novel therapeutic approaches or preventive strategies.

In this systematic review, we aim to comprehensively analyze the available literature on the association between anti-IF and anti-parietal antibodies and *H. pylori* infection in pernicious anemia. By critically evaluating the existing evidence, we seek to identify knowledge gaps, highlight potential mechanisms, and contribute to a better understanding of this complex relationship.

Methods

The systematic review was designed, and the results were reported adhering to the principles set by Preferred Items for Systematic Reviews and Meta-Analysis (PRISMA) guidelines 2020 [9].

Search Strategy and Data Collection

A comprehensive literature search was conducted using multiple databases including PubMed, PubMed Central, and Google Scholar. The search strategy involved utilizing relevant keywords such as "pernicious anemia," "cobalamin," "gastritis," "autoimmune gastritis," "*Helicobacter pylori*," and "intrinsic factor antibodies." Boolean operators such as "AND" and "OR" were used to combine the keywords effectively and broaden the search scope. To further refine the search results, subject-specific Mesh terms were incorporated, ensuring a more targeted approach. The selection criteria for articles focused on the relationship between *H. pylori*, AIG, and pernicious anemia. Similar keywords and terms were applied to other databases, expanding the search to encompass a wider range of scholarly resources.

This comprehensive search strategy aimed to retrieve relevant articles that explore the association between pernicious anemia, AIG, and *H. pylori* infection. By utilizing various databases, such as Pubmed, Pubmed Central (PMC), subject-specific terms, and appropriate search operators, the intention was to obtain a comprehensive and diverse collection of scholarly articles to support the systematic review.

After obtaining the titles, a manual screening was done by considering a parameter of a timeline of 20 years, and titles not fitting the said criterion were excluded. All the references of the papers were meticulously checked for any potentially overlooked publications. The titles, abstracts, and subject headings were also reviewed for relevance. The outcomes were identified, and data were extracted by the corresponding authors and a stringent peer review was performed as well.

Inclusion and Exclusion Criteria

Our inclusion criteria included papers published in the last 20 years, papers pertaining to the research question, papers published in English, and papers with full text. The population group included both pediatric and adult patients with pernicious anemia.

The excluded papers were those that were unrelated to the research topic, published in another language apart from English, and those published over 30 years ago, and papers without full-text access.

Quality Appraisal Tools

To ensure the reliability and validity of the extracted publications, a stringent quality assessment was performed using approved quality appraisal tools for each specific type of study. The Newcastle-Ottawa tool was used to assess the quality of observational studies, while the Joanna Briggs Institute checklist was employed for clinical case reports and expert opinion articles [10]. The non-bias percentage was calculated based on the respective assessment tool. Only studies with a minimum non-bias percentage above 40% were included in the study, ensuring a rigorous selection process and maintaining high-quality standards. This meticulous quality check aims to enhance the credibility and reliability of the included studies, thereby strengthening the overall findings and conclusions of the systematic review.

Results

**Figure 1:**
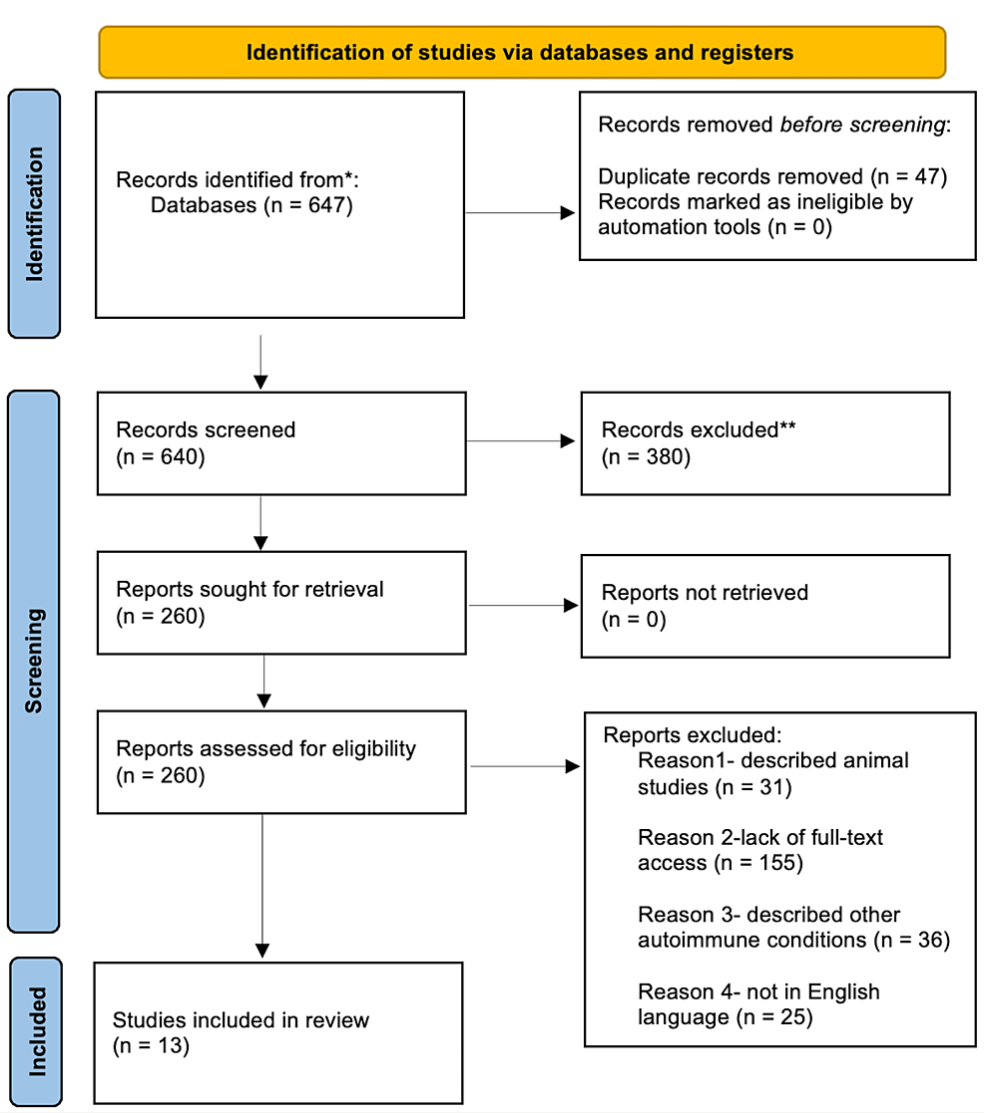
Preferred Reporting Items for Systematic Reviews and Meta-Analyses (PRISMA) flowchart depicting collection and finalizing of studies * Indicates records gathered from databases such as PubMed and PubMed Central (PMC). ** Records excluded after applying a timeline filter of the last 20 years.

The primary search for relevant articles was conducted on PubMed and PMC. A total of 647 articles were initially gathered before a tailored search strategy was applied. Forty articles were immediately removed as they were duplicates. On applying a time filter of 20 years the number of available studies was 260. The inclusion and exclusion criteria were applied to the criterion to refine the search strategy and it resulted in the removal of 211 papers due to a variety of reasons. The citations of the primary selection were then transferred to the EndNote citation manager (EndNote, Clarivate, London, United Kingdom). Thirty-six more papers were deleted by manual scrutiny as they described other autoimmune conditions and through exclusion protocol was implemented to procure papers pertaining to our research topic. A final number of 13 was obtained and these were the studies included in this systematic review. A flowchart description of the search process and selection has been elicited in Figure 1.

## Review

Pernicious anemia is a classic disorder characterized by the impaired absorption of vitamin B12 due to the lack of IF secretion by gastric parietal cells [1]. It is closely associated with AIG, a chronic inflammatory condition that primarily affects the gastric corpus and leads to the destruction of parietal cells [3]. AIG is characterized by the presence of specific antibodies, including anti-intrinsic factor antibodies (AIFA) and anti-parietal cell antibodies (APCA) [3,11]. These antibodies play a crucial role in the pathogenesis of pernicious anemia by inhibiting the binding of vitamin B12 to IF and causing the destruction of gastric parietal cells [3,7].

The understanding of the relationship between pernicious anemia and AIG has evolved over the years. Historical perspectives have shed light on the early recognition and management of pernicious anemia, highlighting the importance of IF deficiency [1,2]. Recent gastroenterological insights have further expanded our understanding of pernicious anemia from a pathophysiological perspective, emphasizing the role of AIG in its development [3].

In this discussion, we will delve into the association between pernicious anemia and AIG, exploring the underlying mechanisms and the significance of specific antibodies in the pathogenesis of pernicious anemia. By examining the current literature, we aim to provide a comprehensive overview of this relationship and its clinical implications. The subsequent parts of the discussion will further explore the association between *H. pylori* infection and AIG, as well as the impact of *H. pylori* eradication on the levels of AIFA and APCA. A summarized table depicting the significant highlights of the selected studies has been shown in Table 1.

**Table 1:** Summary of selected studies Autoimmune gastritis (AIG), anti-parietal cell antibodies (APCA), anti-intrinsic factor antibodies (AIFA), *Helicobacter pylori* (*H. pylori*)

Study	Highlights
Association Between Vitamin B12 Level and Anti-parietal Cells and Anti-intrinsic Factor Antibodies Among Adult Jordanian Patients With Helicobacter pylori Infection [7]	Study demonstrated a statistically significant difference in serum vitamin B12 levels between patients infected and not infected with *H. pylori*, with the infected group showing lower serum vitamin B12 levels (p-value < 0.05).
Insights Into Pediatric Autoimmune Gastritis: Is There a Role for Helicobacter pylori Infection? [8]	No significant statistical differences in terms of hemoglobin levels, gastrin levels, or the presence/grade of oxyntic mucosa atrophy between those with and without the *H. pylori* infection.
Investigations Proposed to Accurately Classify Chronic Gastritis [12]	Expert review describing the classification of *H. pylori*-induced chronic gastritis
Mucocutaneous Manifestations in Autoimmune Gastritis: A Prospective Case-Control Study [13]	Those with AIG were more likely to have concurrent immunological diseases exhibiting mucocutaneous manifestations compared to seronegative individuals (100% vs. 58.5%; P = 0.016).
Helicobacter pylori Infection and Gastric Autoimmune Diseases: Is There a Link? [14]	58% of the parietal cell antibody-positive subjects had *H. pylori* infection (p = .03).
Long-Term Effect of Helicobacter pylori Eradication on Plasma Homocysteine in Elderly Patients With Cobalamin Deficiency [15]	Eradication of *H. pylori* in elderly patients with cobalamin deficiency led to increased cobalamin levels and decreased homocysteine blood levels over a prolonged period.
Atrophic Gastritis and Autoimmunity: Results from a Prospective, Multicenter Study [16]	Higher APCA and AIFA positivity was confirmed in AIG, unrelated to *H. pylori* infection, age, or gender in multivariate analysis.
Rare Case of Pernicious Anaemia From a University Hospital of Nepal: A Case Report [17]	*H. pylori *eradication may have the potential to improve the seropositivity of APCA and AIFA and reverse the manifestations of pernicious anemia
A Multicenter Retrospective Analysis of the Clinical Features of Pernicious Anemia in a Korean Population [18]	7/34 patients (20.6%) tested positive for *H. pylori*. The rate is notably lower than the 60% to 70% positivity typically observed in the broader Korean population
Association of Autoimmune Type Atrophic Corpus Gastritis With Helicobacter pylori Infection [19]	Among 14 patients with severe gastric atrophy and no ongoing *H. pylori* infection, eight had *H. pylori *antibodies detected by immunoblotting. All eight of these patients exhibited elevated APCA, and 4 out of 8 also had AIFA
Oxyntic Gastric Atrophy in Helicobacter pylori Gastritis Is Distinct From Autoimmune Gastritis [20]	Although this study showed an association between *H. pylori* and oxyntic gastric atrophy, we included this study to assess if that led to pernicious anemia.
Healing of Active, Non-Atrophic Autoimmune Gastritis by H. pylori Eradication [21]	*H. pylori* eradication treatment led to the healing of active autoimmune corpus gastritis. Healing was evidenced by the disappearance of gastritis activity, reduction in lymphocytic infiltration, glandular destruction, and parietal cell hypertrophy.
Regression of Autoimmune Gastritis after Eradication of Helicobacter pylori [22]	Coexistence of active *H. pylori* infection and AIG was confirmed through positive APCA at a titer of 1:160, elevated serum gastrin level (638 pg/mL), and positive anti-*H. pylori *antibody (Hp Ab) and *H. pylori* stool antigen tests.
Gastritis [23]	This is merely a narrative article of gastritis and its classification
Impact of Helicobacter pylori on the Development of Vitamin B12 Deficiency in the Absence of Gastric Atrophy [24]	Presented evidence linking *Helicobacter pylori* infection with cobalamin deficiency.

Role of H. pylori and AIG

The role of *H. pylori* in atrophic gastritis has been extensively studied, and its association with AIG has been a topic of interest. In this discussion, we will explore the relationship between *H. pylori *and AIG, based on the findings from various studies.

A large-scale cross-sectional study was performed in Jordan in 2013 to elucidate the association between B12 levels and anti-parietal cells and AIFA [7]. The study reported that 9.9% of *H. pylori*-positive patients had positive APCA, and 18.5% had positive AIFA. In contrast, none of the *H. pylori*-negative subjects had these antibodies. Additionally, *H. pylori*-infected patients exhibited lower vitamin B12 levels (275 ± 70.4 pg/mL) compared to controls (322.9 ± 60.7 pg/mL; p < 0.05). These findings suggest a potential association between *H. pylori* infection and the pathogenesis of AIG.

A study by Silva MH and colleagues where the same association was assessed among the pediatric population concluded with promising results. Among the patients, 18.8% had AIG confirmed by histopathology [8]. Remarkably, none of the AIG patients had evidence of *H. pylori *infection (P = .004). Furthermore, AIG patients had a longer duration of vitamin B12 deficiency (P = .022), lower hemoglobin levels (P = .018), and higher APCA (P = .039) and gastrin (P = .002) levels compared to the non-AIG group. Although over half of the pediatric patients with AIG were found to be *H. pylori* positive, the study did not establish any significant statistical differences in terms of hemoglobin levels, gastrin levels, or the presence/grade of oxyntic mucosa atrophy between those with and without the *H. pylori* infection. This raises questions regarding the direct role of *H. pylori* in the development or severity of AIG in the pediatric population.

A study by Presotto et al. investigated the prevalence of atrophic body gastritis in asymptomatic subjects with APCA [14]. The study found atrophic body gastritis in 14 out of 79 APCA-positive subjects (18%), while only two out of 66 controls (3%) had atrophic gastritis (p = .01). Mean levels of gastrin were increased (p < .0001), while those of pepsinogen were reduced (p < .001) compared to controls. Additionally, *H. pylori* infection was detected in 46 APCA-positive subjects (58%) compared to 26 controls (39%) (p = .03).

Drawing inferences from these studies, the presence of APCA and AIFA in *H. pylori*-positive patients, as observed in the Jordanian study, may suggest a potential link between *H. pylori* infection and AIG [7]. The higher prevalence of AIG in APCA-positive patients, along with the absence of *H. pylori* infection in AIG patients, as demonstrated in the pediatric study, further supports the notion of *H. pylori-*independent mechanisms in the development of AIG [8]. These findings indicate that AIG may involve multiple pathways beyond the influence of *H. pylori*.

Furthermore, the study by Presotto and colleagues [14] reveals a higher prevalence of atrophic body gastritis in APCA-positive subjects compared to controls. The elevated levels of gastrin and reduced levels of pepsinogen in APCA-positive subjects further indicate an association between APCA and the development of atrophic gastritis. The detection of *H. pylori* infection in a significant proportion of APCA-positive subjects suggests a potential role of *H. pylori* in the pathogenesis of atrophic gastritis.

Does Treating H. pylori Reduce the Seropositivity of APCA and AIFA and Reverse Pernicious Anemia?

To reiterate, in a clinical study with 100 participants conducted in Jordan previously discussed above, researchers found a significant association between *H. pylori* infection and the seropositivity of APCA and AIFA [7]. Specifically, 9.9% of *H. pylori*-positive patients exhibited positive APCA, while 18.5% had positive AIFA. These findings emphasize the potential role of *H. pylori* in the pathogenesis of AIG, as evidenced by the presence of specific antibodies.

Furthermore, a study titled "Rare Case of Pernicious Anaemia from a University Hospital of Nepal: A Case Report" highlighted a positive correlation between *H. pylori* infection and pernicious anemia [17]. Although specific statistical values were not provided in the excerpt, this case report suggests that *H. pylori *eradication may have the potential to improve the seropositivity of APCA and AIFA and reverse the manifestations of pernicious anemia. Similar results were found in another study titled "Regression of AIG after *Helicobacter pylori* Eradication: A Case Report" from 2023 January presented a case of *H. pylori*-positive AIG in which eradication therapy resulted in improved histopathological findings and reduced atrophic changes in the gastric corpus [22]. The patient exhibited a decrease in APCA titers from 1:160 to 1:40 and a reduction in *H. pylori* antibody (HpA) titers after seven months of eradication therapy. These findings suggest a potential therapeutic effect of *H. pylori* eradication on AIG.

To further our understanding and caveats of gastritis we assessed a narrative review by Desai HG which spoke about the updated classification of gastritis. In that paper, the author discussed the classification of gastritis based on immunological parameters and the detection of present or past *H. pylori* infection [12]. It emphasized that the presence ofHpA plays a crucial role in determining the progress and localization of gastric mucosal damage, especially in patients with corporal atrophic gastritis. This indicates the complex relationship between *H. pylori* infection, AIG, and the progression of gastric mucosal damage. The expert opinion from the above commentary resonated in another similar study titled "Healing of Active, Non-atrophic AIG by *H. pylori* Eradication" that investigated the effect of *H. pylori* eradication treatment on autoimmune corpus gastritis [21]. The study revealed that *H. pylori* eradication led to the healing of active autoimmune corpus gastritis, as evidenced by reduced activity of gastritis, lymphocytic infiltration, glandular destruction, and parietal cell hypertrophy.

In accordance with the studies discussed above, we came across a large-scale observational study conducted by Serin E et al., in Turkey which explored the association between *H. pylori* infection, serum vitamin B12 levels, and histopathological changes in patients with minimal or no gastric atrophy [24]. The study demonstrated an inverse correlation between serum cobalamin levels and histopathological scores, indicating that lower vitamin B12 levels were associated with more severe histopathological changes. Additionally, improvements in hematologic parameters and serum vitamin B12 levels were observed after *H. pylori* eradication, regardless of the eradication status. Another study conducted on adults showed that *H. pylori* eradication therapy in elderly patients with cobalamin deficiency effectively resulted in decreased homocysteine levels and increased cobalamin levels [15]. These findings suggest that *H. pylori* eradication may contribute to the normalization of blood parameters associated with cobalamin deficiency.

By analyzing and comparing the findings from these studies, it becomes evident that *H. pylori* infection is associated with higher seropositivity rates of APCA and AIFA, lower vitamin B12 levels, and more severe histopathological changes. The eradication of *H. pylori* appears to have potential therapeutic benefits in terms of reducing APCA and HpA titers, improving histopathological findings, and reversing atrophic changes in the gastric corpus. However, it is important to note that the specific numerical values and statistical analyses varied across the studies, making direct comparisons challenging.

Limitations

One major limitation of this study we felt was the dearth of observational studies; with the availability of more case-control studies, research on this topic can be more robust and improve clinical research outcomes. Another possible limitation was the exclusion of over 100 articles due to lack of full-text access; the implications of which are oblivious to the research team.

## Conclusions

In conclusion, these studies provide valuable insights into the relationship between *H. pylori* infection, seropositivity of APCA and AIFA, and the development of pernicious anemia and AIG. Although further research is warranted to establish definitive conclusions and guide clinical management strategies, the evidence suggests a potential role of *H. pylori* in the pathogenesis of these conditions. The findings highlight the importance of considering *H. pylori* infection in the evaluation and management of patients with pernicious anemia and AIG, as eradication therapy may have beneficial effects on the serological markers and histopathological changes associated with these conditions.
